# The Effect of Spiritual Intelligence Training on Job Satisfaction of Psychiatric Nurses

**Published:** 2017-04

**Authors:** Abbas Heydari, Ali Meshkinyazd, Parvaneh Soudmand

**Affiliations:** 1 Department of Medical – Surgical Nursing, School of Nursing and Midwifery, Mashhad University of Medical Sciences, Mashhad, Iran.; 2 Department of Nursing and Midwifery, Mashhad University of Medical Sciences, Mashhad, Iran.

**Keywords:** *Spiritual Intelligence*, *Psychiatric Nurses*, *Job Satisfaction*, *Psychiatric Ward*

## Abstract

**Objective:** Nurses are the most important staff in the health care system, thus, their job satisfaction is important in nursing management. The present study aimed at determining the impact of teaching spiritual intelligence on the job satisfaction of psychiatric nurses.

**Method:** The participants were divided into 2 groups by random allocation. Data were collected in 3 stages of before intervention, 4 weeks, and 8 weeks post intervention using Brayfield & Rother Job Satisfaction Questionnaire.

**Results:** The results of this study revealed that the mean score of job satisfaction in the experimental group was 65.5±9.9 in the pre intervention stage, which increased to 69.8±6.3 one month after the intervention and to 72.5±8.9 in 2 months after the intervention, and it was significantly more than that of the control group.

**Conclusion:** The job satisfaction rate of the control group decreased admirably in both 1 month and 2 months after the intervention stage. Thus, spiritual intelligence training is an effective method to increase job satisfaction, and it is suggested that managers consider spiritual intelligence training to increase job satisfaction in nurses.

In the recent years, much attention has been given to job satisfaction as a key concept in nursing research. This extensive and considerable attention shows the huge impact of job satisfaction on nurses, patients, hospitals, and the nursing profession (1). Job satisfaction is a positive and emotional state derived from individual's assessment of their job or job experience (2). Because nurses have a major role in providing healthcare services in hospitals, factors that influence their job satisfaction directly affect the quality of care provided to patients (3). A large number of nurses are leaving their job, and this has become a major global concern. Studies show that job dissatisfaction is one of the most important factors in job quitting; for instance, about one third of nurses in the UK and more than one fifth of US nurses have the intention to leave this profession (4). Many studies have been conducted on job satisfaction in nurses, all of which show that job satisfaction in nursing is very important. In a study by Vaghar et al. (2005), it was concluded that most nurses (88%) who participated in the study were dissatisfied with their occupation and only 1.6% were satisfied (5)

Akhtari- et al. (2004) reported that 72% of nurses were not satisfied with their job (6). Veys Moradi (2007) suggested that most nurses (54%) working in internal and surgical wards had low job satisfaction (7).

Published researches in different countries present that job satisfaction is a major predictive factor for job absences, job burnouts, turnovers, and resignations among nurses (4). Psychiatric hospital wards are highly stressful because nurses continually face stressful and exhausting stimulants (8, 9). According to the results of various studies, about half of nurses working in psychiatric wards experience excessive stress and emotional exhaustion (10). The studies show that stress is not only a risk to nurses’ health, but it also extremely influences their ability to deal with it (11). Considering the crucial role of nurses in providing care and meeting the patients’ needs, it is essential to pay attention to nurses’ job satisfaction and try to enhance it. If nurses are motivated enough, they can provide better services and be more efficient in their occupation. Thus, as stress is an influential factor in job satisfaction of the nurses, it is necessary for psychiatric nurses to have enough knowledge of relaxation and stress management. Therefore, stress management programs can probably be beneficial in improving their job satisfaction, and consequently, maintaining and improving the quality of care. Evidence suggests that psychological therapies (including meditation, music-therapy, massage, cognitive-behavioral stress management) are effective in reducing and controlling stress. (12). McFarland suggests that strategies such as coping with teamwork, extracurricular activities, humor, positive feedback from managers, and participation in stress management programs have been helpful in reducing the stress of nurses and physicians (13). Douglas et al. reported that nurses use leisure activities, visiting friends, and family and relaxation to cope with stressors (14). Studies showed that there is a high correlation between spiritual intelligence with the purpose of life, life satisfaction, job stress, and mental health. According to George, important characteristics of spiritual intelligence include personal confidence, effective communication, interpersonal understanding, managing changes, and move from difficult routes. Spiritual intelligence is one of the multiple intelligences that can independently grow and develop (15). According to Zohar and Marshall, spiritual intelligence allows a person to gain a deep insight into events of life, avoid fear of hardships in life, confront them with patience, and find rational and humane solutions (16). Spiritual intelligence increases flexibility and consciousness against difficulties and hardships of life (17). In fact, spiritual intelligence is a set of activities beside gentleness and flexibility in behavior, which causes individual consciousness and deep insights towards life and its purpose so that goals could be traced beyond the material world. This process requires the individual's adaptation to the environment because of the satisfaction of others (18). In George's opinion, the most important application of spiritual intelligence in the workplace is to create peace of mind, and mutual understanding among colleagues (15). The study conducted by Karimi et al. revealed that about 45% of nurses have spiritual intelligence scores lower than the average (19). Due to the importance of job satisfaction in providing quality care to patients and considering the fact that simply a single study cannot find the effect of spiritual intelligence training on psychiatric nurses’ job satisfaction, the researchers decided to conduct this research.

## Materials and Methods

***Procedure***

This experimental study was conducted on psychiatric nurses working in the psychiatric hospital of Mashhad, Iran, in 2015. This hospital was selected as the study setting for its size (the largest psychiatric hospital in the East of the country, with 750 active beds). Study inclusion criteria were as follow: willingness to participate, BSc or MSc in nursing, at least 6 months work experience in Ibn-Sina hospital, no use of psychotropic medication, and no more than 2 weeks of absence in the last month. Study exclusion criteria were as follow: unwillingness to participate, absence in more than 10% of the workshop time, leaving or being transferred to another hospital, and exposure to major stressors (death of relatives, divorce etc.) in the course of the study. Arrangements were made with the education supervisor for the nurses’ enrollment in the spiritual intelligence workshop, and related posters were posted in various hospital departments.

***Participants***

, 60 nurses voluntarily enrolled the study. Participants were randomly divided into intervention ( n = 30) and control (n = 30) groups according to their enrollment code (odd numbers as the intervention, and even numbers as controls). Due to withdrawal of 6 nurses (3 for non-participation in the workshop, 1 for sick leave, and 2 for incomplete questionnaire answering in the follow-up stage), 54 nurses participated in the study (27 in each group).

***Materials***

Tools used in this study included a personal and occupational details form, and Brayfield and Rothe Questionnaire of Job Satisfaction. The demographic questionnaire contained 16 items about personal and occupational issues, and was prepared according to study objectives and review of the latest literature and articles. The Job Satisfaction Questionnaire contains 19 items, and respondents were asked to identify their feelings and attitude towards their job according to a 5-point scale (totally disagree, disagree, undecided, agree, and totally agree). Items 5, 9, 7, 11, 12, 15, 17, and 19 are inversely scored. The overall score of this tool ranges from 19 to 95. Higher overall scores indicate higher job satisfaction. Validity of this questionnaire was confirmed by 10 experts from Mahshad School of Nursing and Midwifery. Reliability of the Persian version was found by Abdollahzadeh & Kermorodi using internal consistency with Cronbach's alpha of 0.93 (20). In this study, reliability of job satisfaction questionnaire was found using internal consistency with Cronbach's alpha 0.88. Data were collected simultaneously from both groups before, 1 month after, and 2 months after the intervention.

***Intervention Protocol***

Approval was obtained by the Research Council of Mashahd University of Medical Sciences and the ethics committee in 2015. Then, spiritual intelligence training began for the intervention group as 7 weekly sessions in 2 months using spiritual intelligence training protocol based on Zohar and Marshall (16), Emmons (17) and Sisk and Torrance (21), where each session took 90 minutes. The implementation of the sessions is described in Table 1. The control group received a lecture by the researcher on psychiatric signs and symptoms and common terminologies in Ibn-Sina hospital amphitheater over two 3-hour sessions with a week interval. Participants were assessed 1 and 2 months after the intervention. During this time, researchers contacted the participants in the intervention group every 2 weeks by phone to follow-up and reinforce compliance with the educational program.

***Statistical*** ***Analysis***

Data were analyzed in SPSS-19 software. Normal distribution of quantitative variables was confirmed using Kolmogorov-Smirnov test. Demographic and personal details were described using mean, standard deviation, and percentages. A Chi-square test was used to confirm the 2 groups matched in qualitative variables, and normally distributed quantitative variables were compared using t test (and Mann-Whitney test, if not normally distributed). T test was also used to compare the 2 groups for job stress (and Mann-Whitney test, if not normally distributed). Repeated analysis of variance and Friedman test were used for intragroup comparison. All the above tests were conducted at the confidence interval 95% and significance level of 0.05. 

## Results

In this experimental study, of the 54 participating nurses, 74.1% in the intervention group and 55.5% in the control group were males, with a mean age of 33±4.1 years. Of all participants, 85.2% from the intervention group and 77.7% from the control group were married, and 96.3% in each group had BSc in nursing. Participants' mean psychiatric work experience was 6.8±4.4 years. The majority of the participants in both groups (70.4% in the intervention group and 77.8% in the controls) were in contract employment terms. The 2 groups matched in all background variables, with no significant differences between them (Table 2). In the intervention group, the mean and standard deviation of job satisfaction scores increased from 65.5±9.9 before the intervention to 69.8±6.3 after the intervention and 72.5±8.9 at the follow-up one month later. In the control group, mean job satisfaction score decreased from 64.4±9 before the intervention to 58.5±3.2 after the intervention and 56.5±9.6 at the follow-up stage one month later. There were no significant differences between the 2 groups in job satisfaction before the intervention (P = 0.63). Independent t test results showed a significant difference between the 2 groups in mean job satisfaction score after the intervention, where the intervention group score showed a significant increase compared to that of the control group (Table 3).

In the intervention group, comparing mean stress before, 1 month and 2 months after the intervention (intragroup comparison), using repeated measures of ANOVA, showed significant differences between the 3 stages (P = 0.000). In the control group, repeated measures ANOVA results (intragroup comparison) revealed significant differences in the mean stress among the 3 stages (P = 0.000). This test showed a significant difference among these stages (P = 0.000).

**Table1:** Spiritual Intelligence Components Training Protocol

**Session**	**Subject**
Session 1	Introducing the overall structure of the sessions, and expressed expectations, and regulations
Session 2	Training in the field of increasing self-consciousness (existing relationships with the transcendent, other people)
Session 3	Training in the field of honesty with yourself and practice in accordance with the guidance in the field of education
Session 4	Training in the field of the intuitive meaning and its role in life (a mission in life, a sense of holiness in life)
Session 5	Training in the field of addressing ethical issues and the importance of giving them love, compassion, humility, kindness, forgiveness and healing, link, and creative service
Session 6	Training in the field of their capabilities to control themselves, and behave kindly, and empathetically regardless to the circumstances
Session 7	Training in the field of the concept of flexibility and its necessity in everyday life

**Table 2:** Demographic Characteristics in Teaching Spiritual Intelligence and Control

**Group**			
**Variable **	**Teaching ** **spiritual ** **intelligence**	**Control**	**Test results**
	The number (percentage)	The number (percentage)	
Age (years)	33.0±4.3	32.0±4.2	T = -0.412 Df = 52 p-_value_ =0.682
Marriage single Married	(22.3) 6	(14.8)4	Chi-square = 0.491 Df = 1 p-_value = 0.484_
	(77.7)21	(85.2)23	
Education level BS MC	( 96.3)26	(96.3)26	Fisher Exact test = 1.000
	(3.7)1	(3.7)1	
Employment status official Contractual	(22.2)6	(29.6)8	Chi-square = 0.386 Df = 1p-_value = 0.535_
	(77.8)21	(70.4)19	

**Table 3:** Comparison of the Mean Score of Nurses' Job Satisfaction in Psychiatric Wards in the Two Groups Before the Intervention, One Month After the Intervention, and at Follow-up Two Months Later

**Group**			
	**Teaching spiritual ** **intelligence**	**Control**	**Independent t ** **test**
	**mean** **±** **SD**	**mean** **±** **SD**	
Before intervention	65.5**±**9.9	64.4**±**9/0	p=0.630
One month after intervention	69.8**±**6.3	5 5.8**±**3.2	p=0.030
At the 2-month follow-up	72.5**±**8.9	5 6.5**±**9.6	p=0.000
	p=0/000	p=0.000	

## Discussion

This study revealed that significant improvement in average job satisfaction score in the intervention group after the intervention proves the positive effect of spiritual intelligence education on job satisfaction of psychiatric nurses. Because no similar study was found to compare the effect of spiritual intelligence education on job satisfaction, the results of the present study were compared to those studies that aimed at determining the effects of life skills interventions or cognitive-behavioral ones on job satisfaction. Proudfoot assessed the effect of cognitive-behavioral intervention on job satisfaction in employees of an insurance company in England, and their results showed a significant increase in average job satisfaction score in the intervention group compared to the control group following 7 intervention sessions (22). Their results on the impact of cognitive - behavioral interventions on job satisfaction were similar to those of the present research. Thus, as internal sense of empowerment increases, so does job satisfaction (23). In the present study, the effect of spiritual intelligence training on job satisfaction of nurses increased at the follow-up stage 1 month later compared to the time of the intervention. This was probably due to the phone contact made by the researchers to the participants of the intervention group once every 2 weeks to follow up and reinforce compliance with the education program. In addition, at the follow-up period, nurses had the opportunity to practice skills learnt in real life, and internalize this program in their daily lives by constant follow-up and practice, which led to increased effect of the intervention over time. Durban et al. in their study assessed the effect of stress immunization program on job satisfaction in psychiatric nurses. Their results showed significantly greater mean job satisfaction in the intervention group compared to the controls after 2 intervention sessions (24), which agrees with the results of the present study.

The results of this study revealed that the lowest level of job satisfaction in the control group was at the follow-up, indicating that over time, not only job satisfaction did not improve, but also it decreased. This may be attributed to the reducing physical and mental strength of nurses every day, adversely affecting their job satisfaction. Teaching psychiatric symptoms and common terms to the control group participants had no effect on their job satisfaction. Hence, increased job satisfaction in the intervention group must have been due to the education they received.

## Limitations

In the present study, the most important limitations were as follow:

1-           Small sample size and selection of participants from 1 hospital, which limits the generalizability of the results

2-           Personal differences, psychological characteristics, and personal and family attributes affecting answers to questions and effectiveness of workshop, which were matched in the 2 groups as much as possible through random allocation of participants to groups

3-           Lack of complete control over information exchange between groups; To reduce spread of data, groups were matched in their working shifts and post. In addition, the intervention group was asked to refrain from spreading data until the end of study, and the control group was assured of participating in the spiritual intelligence workshop after the end of intervention. Thus, this way, exchange of data was largely controlled. However, it may have slightly occurred beyond control.

## Conclusions

In the healthcare system, nurses are the main health service-providing group, and accordingly, they should have high levels of job satisfaction to enable them to provide high quality care to patients. This is possible, when nurses are in a satisfactory mental state. Therefore, attention should be paid to nurses' health and job satisfaction to retain the nursing workforce as humans in the first place, and later as those who provide health care services to other members of the community. The results revealed that spiritual intelligence education workshop can increase job satisfaction in nurses, and it can be used as a strategy to reduce job stress in nurses.
